# Post-maceration storage temperature affects sperm quality and fertilization in sex-reversed rainbow trout

**DOI:** 10.1038/s41598-026-46962-4

**Published:** 2026-04-11

**Authors:** Sebastián Ávila, Osvaldo Merino, Leydy Sandoval-Vargas, Francisco Estay, Miguel Colihueque Mancilla, Paola Niedmann, Eduardo Rojas, Wellison Amorim Pereira, Iván Valdebenito Isler, Elías Figueroa-Villalobos

**Affiliations:** 1 Doctorado en Ciencias Agropecuarias, Facultad de Recursos Naturales, Universidad Católica de Temuco, Temuco, Chile; 2 Núcleo de Investigación en Producción Alimentaria, Facultad de Recursos Naturales, Universidad Católica de Temuco, Temuco, Chile; 3 Center of Excellence of Biotechnology in Reproduction (BIOREN-CEBIOR), Faculty of Medicine, University of La Frontera, Temuco, Chile; 4 Piscícola Huililco Ltda, Pucón, Chile; 5 Laboratory of Microbial Biomolecules, School of Pharmaceutical Sciences, University of São Paulo, São Paulo, Brazil; 6 Center for Research, Innovation and Creation (CIIC-UCT), Catholic University of Temuco, Temuco, Chile; 7 Departamento de Ciencias Agropecuarias y Acuícolas, Facultad de Recursos Naturales, Universidad Católica de Temuco, Temuco, Chile

**Keywords:** Aquaculture, Fish reproduction, Sex-reversed male, Thermal stress, Sperm quality, Flow cytometry, Biochemistry, Biological techniques, Biotechnology, Cell biology, Physiology, Zoology

## Abstract

Optimizing sperm quality is essential in aquaculture, especially for producing all-female *Oncorhynchus mykiss* populations using sex-reversed males. Because sex-reversed males lack sperm ducts, semen is collected by gonadal maceration, a step that may expose spermatozoa to thermal and mechanical stress. We evaluated how post-maceration storage temperature (2, 4, 12 °C for 24 h) is associated with sperm performance in a within-male design, using an immediate post-extraction control (T0, 0 h) as a descriptive baseline for comparison. Sperm kinematics were quantified by computer-assisted sperm analysis, and cellular readouts were assessed by flow cytometry for viability (% SYTOX-negative), mitochondrial membrane potential (ΔΨm/% JC-1 red), superoxide anion production (DHE/% DHE-high), and DNA fragmentation (% TUNEL-positive). After 24 h, the warmer condition (12 °C) was associated with lower total and progressive motility, lower mitochondrial membrane potential and viability, and higher superoxide levels, relative to cold conditions (2–4 °C). Fertilization was analyzed as binomial data (k/n); proportions were lower at 12 °C, but should be interpreted with caution given the small number of eggs assessed per replicate. Correlations between cellular biomarkers and motility/fertilization were generally weak, although a moderate negative association was observed between mitochondrial status and superoxide anion production. Overall, these results suggest that sperm from sex-reversed males is sensitive to post-maceration handling temperature, supporting the practical approach of maintaining tissues, tools and homogenates at ~ 0–4 °C throughout the collection and storage workflow.

**Supplementary Information:**

The online version contains supplementary material available at 10.1038/s41598-026-46962-4.

## Introduction

Reproductive efficiency is a fundamental pillar in fish farming today, especially in intensive systems where uniform growth, controlled reproduction, and genetic selection programs are vital to ensure economic viability. In salmonid farming, producing all-female populations of *Oncorhynchus mykiss* (rainbow trout) has become a widely used approach to prevent early male maturation and promote consistent harvests^1,2^. This is made possible through the creation of sex-reversed males (genetic females) that are hormonally induced to develop testes and produce spermatozoa containing only X chromosomes^3,4^. These individuals, known as “sex-reversed males”, are indispensable for the production of monosex progeny; however, their reproductive use is technically challenging due to their lack of sperm ducts, which necessitates testicular maceration and sacrifice of the fish for semen retrieval^5,6^. In some species, however, and under certain hormonal conditions, the formation of functional gonoducts has been reported in a percentage of individuals, allowing sperm collection through induced spawning or conventional stripping^7,8^.

Unlike conventional stripping methods used to collect sperm, testicular maceration imposes a unique set of stressors on sex-reversed male broodstocks. The process involves not only mechanical tissue disruption but also potential exposure to suboptimal or fluctuating temperatures during handling, conditions that may impair the function of the sperm before it is even used for fertilization^9,10^. This is particularly concerning given the economic and genetic value of sex-reversed males, whose contribution to monosex production relies on the quality of sperm obtained post-mortem. As such, gaining a deeper understanding of how environmental factors, such as post-maceration storage temperature, impact sperm physiology is essential to enhance reproductive outcomes and reduce losses in hatchery operations.

Among the various environmental factors that influence reproduction in ectothermic animals, temperature stands out as one of the most critical. It regulates key physiological processes such as gametogenesis, hormone production, spermiation, and even gamete activation^11,12^. When temperatures stray from the optimal range, sperm cells can lose their homeostatic balance. This may trigger oxidative stress, impair mitochondrial function, and lead to subtle but harmful structural changes. Interestingly, fish sperm exposed to heat showed a marked drop in mitochondrial membrane potential (ΔΨm) even without visible plasma membrane damage, highlighting how early signs of cellular distress can go unnoticed if we rely solely on traditional viability markers^13^. In most hatchery operations, sperm motility is still considered the gold standard for assessing sperm quality, mainly because it is quick, easy to measure, and does not require complex equipment^14^. However, reliance on motility alone can be misleading. Studies have shown that spermatozoa with normal motility may register molecular or organelle-level damage, particularly when subjected to stressors such as heat or oxidative imbalance^2,15^. Hence, the integration of contextual cellular measures capable of detecting early physiological impairments has become a focus of research into reproductive physiology and gamete management.

Among these biomarkers, ΔΨm and intracellular reactive oxygen species (ROS) levels have proven to be particularly informative. ΔΨm, commonly assessed by flow cytometry using JC-1 or Mitotracker dyes, provides a proxy for mitochondrial energetic status, which is crucial for sustaining sperm motility and fertilization capacity^16,17^. On the other hand, ROS levels, especially superoxide anion (O_2_⁻·) production, detectable using SYTOX/DHE fluorescence indicate redox imbalance and are tightly linked to lipid peroxidation, DNA fragmentation, and apoptotic signaling^18,19^. The overproduction of ROS under heat stress conditions may also coincide with reduced activity of antioxidant enzymes in seminal plasma, amplifying cellular vulnerability^10^.

Particularly in sex-reversed males, whose sperm is obtained under suboptimal and invasive conditions, such biomarkers may provide additional physiological insights that conventional metrics lack when evaluating handling stress. Recent studies have confirmed that ΔΨm and ROS levels correlate with sperm viability and fertilization success under cold storage or cryopreservation conditions^6,20^. Moreover, oxidative stress is increasingly recognized as a primary cause of functional sperm impairment in salmonids^21,22^. In the context of global climate variability and increasing hatchery intensification, the need for robust, field-applicable biomarkers is more pressing than ever.

Despite their potential, these biomarkers have been underexplored in the specific case of sex-reversed males, a serious oversight given the increasing reliance on these individuals for all-female production systems. Additionally, spermatozoa from sex-reversed males are particularly sensitive due to their testicular origin, lacking the full maturation implied by emission via the sperm duct^6,23^. As a result, they tend to exhibit higher physiological fragility and non-uniform cellular characteristics during handling.

The objective of this study was to evaluate the effect of different post-maceration storage temperatures (2 °C, 4 °C and 12 °C for 24 h) on sperm quality in sex-reversed males of *O. mykiss*. In addition to traditional metrics such as motility and fertilization rate, we applied flow cytometry to assess key functional biomarkers of sperm quality, including mitochondrial membrane potential, superoxide anion production, plasma membrane integrity, and DNA fragmentation. We hypothesize that the temperature during post-maceration short-term storage alters key physiological parameters of spermatozoa in sex-reversed *O. mykiss*. Our primary comparisons focus on the effects of 24 h storage at different temperatures (T1–T3). Specifically, we propose that functional biomarkers were evaluated to explore differential cellular responses to thermal stress, aiming to provide physiological context to the observed changes in motility and fertilization capacity.

## Results

The effects of maceration temperature and short-term storage on sperm quality were first assessed by evaluating motility parameters. Overall, sperm motility was better preserved at cooler storage temperatures (2–4 °C) compared to the warmer condition (12 °C) after 24 h.

### Sperm quality parameters

#### Motility (CASA)

**Fig. 1:**
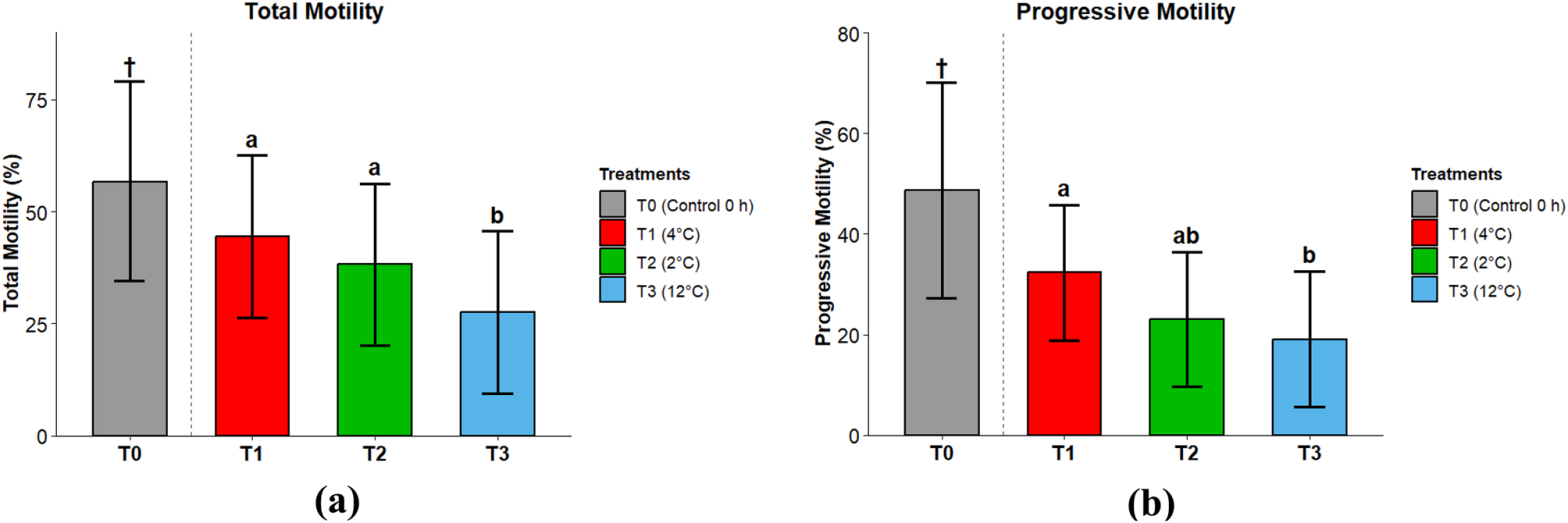
(**A**) Total motility (TM) of sperm from sex-reversed *Oncorhynchus mykiss* under different temperature treatments. Data are presented as Estimated Marginal Means (EMMs) ± 95% confidence intervals. Statistical comparisons (letters) are based on a linear mixed-effects model among T1–T3 only. T0 (†, 4 °C, 0 h) is shown as a descriptive baseline only and was not included in the statistical inference. Acquisition: 3 independent activations, read at 5 s post-activation; ≥ 100 tracks/field; stage at 4 °C. n = 10 sex-reversed males. (**B**) Progressive motility (PM) of sperm from sex-reversed *Oncorhynchus mykiss* under different temperature treatments. Data are presented as Estimated Marginal Means (EMMs) ± 95% confidence intervals. Statistical comparisons (letters) are based on a linear mixed-effects model among T1–T3 only. T0 (†, 4 °C, 0 h) is shown as a descriptive baseline only and was not included in the statistical inference. PM was defined as STR ≥ 45% with VAP ≥ 30 µm s⁻^1^. Acquisition: 3 independent activations, read at 5 s post-activation; ≥ 100 tracks/field; stage at 4 °C. n = 10 sex-reversed males.

We defined total motility (TM) as the percentage of spermatozoa with a VAP ≥ 15 μm·s⁻^1^, while progressive motility (PM) as STR ≥ 45% with VAP ≥ 30 μm·s⁻^1^ (detailed settings in Supplementary Table S1). At 0 h (baseline, 4 °C), total motility averaged 56.7% (95% CI 34.5–79.0%), serving as a reference for the initial condition before storage. Following 24 h of incubation, temperature significantly affected motility patterns (Fig. 1A). Total motility was maintained at comparable levels between 4 °C (T1; 44.5%, 95% CI 26.4–62.5%) and 2 °C (T2; 38.3%, 95% CI 20.2–56.3%), whereas storage at 12 °C (T3) resulted in significantly lower motility (27.6%, 95% CI 9.5–45.7%) compared to the 4 °C treatment (*p* < 0.05). A similar trend was observed for progressive motility (Fig. 1B), where the highest values were recorded at 4 °C (T1; 32.3%, 95% CI 18.9–45.8%). This was significantly higher than the 12 °C condition (T3; 19.1%, 95% CI 5.7–32.6%), while values at 2 °C (T2; 23.0%, 95% CI 9.6–36.5%) were intermediate and not statistically different from the other groups. Kinematic parameters (VCL, VSL, VAP) showed a clear stratification; unlike total motility, velocities were significantly higher at 4 °C compared to both 2 °C and 12 °C, with the latter showing the marked lowest velocities across all metrics *(p* < 0.05; Table 1).

**Table 1:** Kinetic parameters of sperm from sex-reversed rainbow trout (*Oncorhynchus mykiss*) subjected to different storage temperatures.

Group	VCL (μm·s^-1^)	VSL (μm·s^-1^)	VAP (μm·s^-1^)
Control (4 °C, 0 h)	90.7 (74.6–106.9)	63.2 (54.0—72.3)	71.6 (60.0–83.2)
T1 (4 °C, 24 h)	92.0^a^ (79.7–104.3)	62.7^a^ (53.9–71.6)	74.4^a^ (64.3–84.4)
T2 (2 °C, 24 h)	75.1^b^ (62.8–87.5)	48.8^b^ (39.9–57.7)	59.8^b^ (49.7–69.8)
T3 (12 °C, 24 h)	41.0^c^ (28.7–53.3)	22.3^c^ (13.4–31.2)	28.5^c^ (18.5–38.5)

Data are presented as Estimated Marginal Means (EMMs) and 95% confidence intervals (in parentheses). Statistical comparisons are based on a linear mixed-effects model. Different superscript letters (a, b, c) indicate significant differences between treatments (*p* < 0.05). **†** T0 (Control, 0 h) serves as a descriptive baseline and was not included in the inferential analysis. n = 10 males.

### Cellular biomarkers and fertilization rate

**Fig. 2:**
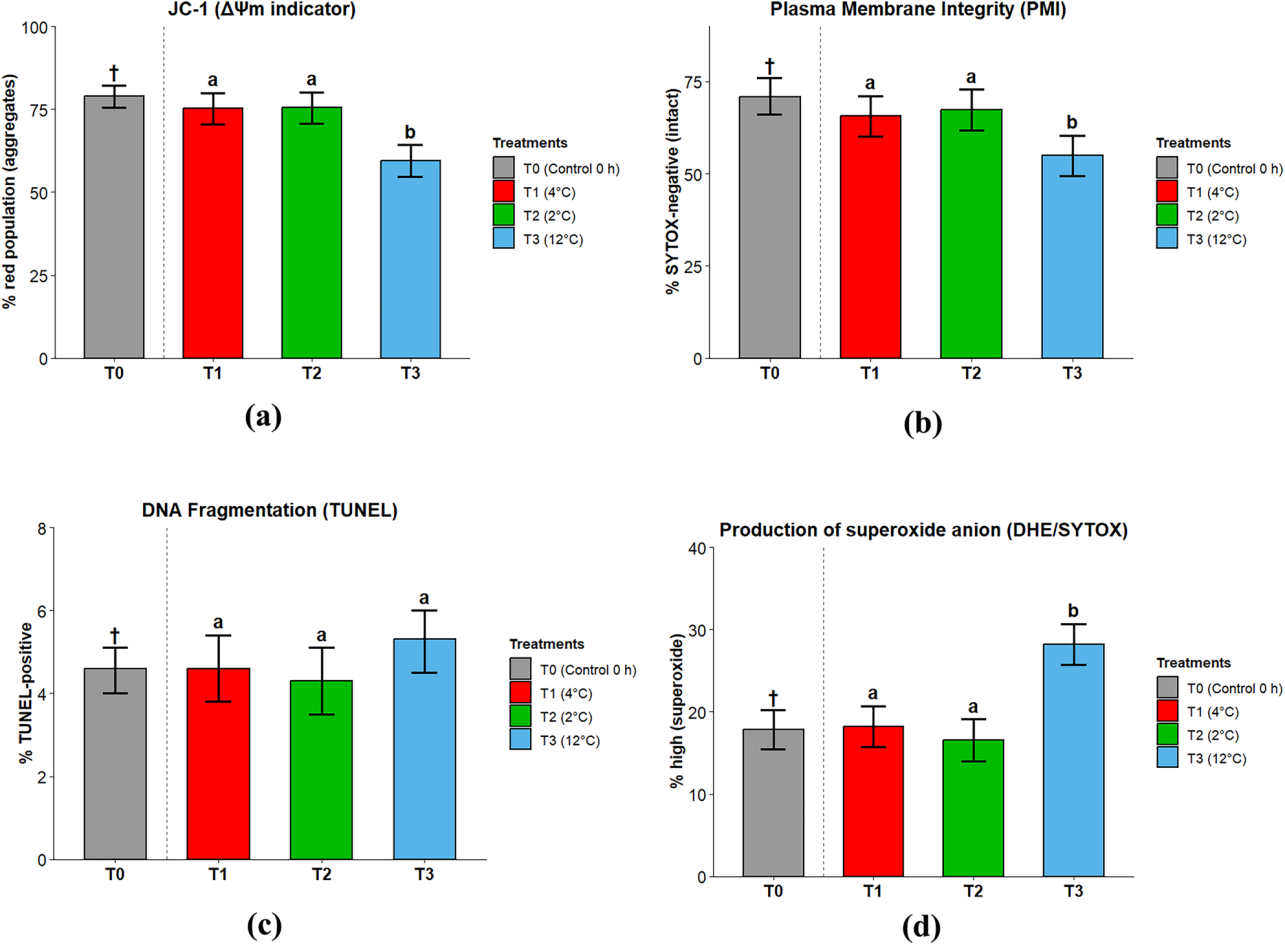
(**A**) JC-1 (ΔΨm). Data are presented as Estimated Marginal Means (EMMs) ± 95% confidence intervals. T0 (†) is descriptive (0 h at 4 °C) and excluded from inference. Letters over T1–T3 indicate significant differences based on a linear mixed-effects model (fixed: treatment; random: male). Endpoint expressed as % red population (aggregates). Acquisition at ~ 4 °C; ≥ 10,000 events/sample. n = 10 sex-reversed males. (**B**) Plasma membrane integrity. Endpoint expressed as % SYTOX-negative (intact). Data are presented as EMMs ± 95% confidence intervals. T0 (†) is descriptive only. Letters over T1–T3 indicate significant differences based on a linear mixed-effects model (fixed: treatment; random: male). Acquisition at ~ 4 °C; ≥ 10,000 events/sample. n = 10 sex-reversed males. (**C**) DNA fragmentation (TUNEL). Endpoint expressed as % TUNEL-positive (gating). Data are presented as EMMs ± 95% confidence intervals. T0 (†) is descriptive only. No significant differences were detected among T1–T3 (linear mixed-effects model; all labeled 'a'). Read at ~ 4 °C; ≥ 10,000 events/sample. n = 10 sex-reversed males. (**D**) Reactive oxygen species. Endpoint reported as % DHE-high (superoxide) defined vs controls. Data are presented as EMMs ± 95% confidence intervals. T0 (†) is descriptive only. Letters over T1–T3 indicate significant differences based on a linear mixed-effects model (fixed: treatment; random: male). Read at ~ 4 °C; ≥ 10,000 events/sample. n = 10 sex-reversed males.

At 0 h (baseline, 4 °C), the proportion of JC-1-high (ΔΨm) cells averaged 78.9% (95% CI 75.5–82.2%), reflecting the initial mitochondrial potential prior to storage. After 24 h, mitochondrial activity varied markedly with temperature (Fig. 2A). Sperm incubated at 12 °C (T3) displayed a lower proportion of JC-1-high cells (59.5%, 95% CI 54.7–64.2%) relative to those kept at 2 °C (T2; 75.5%, 95% CI 70.7–80.2%) and 4 °C (T1; 75.2%, 95% CI 70.4–79.9%) (*p* < 0.05). Plasma membrane integrity (PMI; % SYTOX-negative) showed a similar pattern (Fig. 2B). Samples held at 12 °C had the lowest PMI (T3; 54.9%, 95% CI 49.4–60.4%), significantly below the values at 2 °C (67.3%, 95% CI 61.8–72.8%) and 4 °C (65.6%, 95% CI 60.1–71.1%) (*p* < 0.05). DNA fragmentation, assessed by the TUNEL assay, remained stable across storage temperatures (Fig. 2C), averaging 4.3–5.3% across T1–T3, with no significant differences (*p* > 0.05). In contrast, the proportion of DHE-high sperm (% superoxide) increased markedly under the warmest condition (12 °C; 28.2%, 95% CI 25.7–30.7%), relative to both 2 °C (16.6%, 95% CI 14.0–19.1%) and 4 °C (18.2%, 95% CI 15.7–20.7%) (*p* < 0.05) (Fig. 2D).

### Fertilization (binomial k/n by treatment and male)

**Fig. 3:**
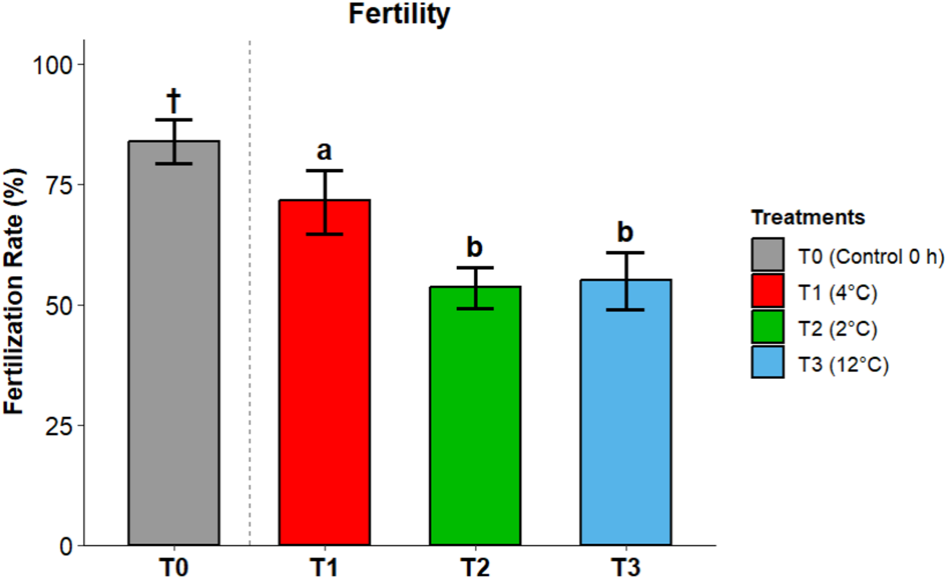
Fertilization rates by treatment. Bars represent Estimated Marginal Means (EMMs) ± 95% confidence intervals derived from a binomial GEE model. Statistical inference (letters denoting significant differences) compares T1–T3 only. T0 (†) is shown descriptively as a baseline reference. n = 10 sex-reversed males; 3 replicates × 20 eggs per male per treatment. Per-male k/n and model summaries are provided in Supplementary Table S-Fert and Table S-Effects.

At 0 h (baseline, 4 °C), fertilization rates averaged 83.8% (95% CI 79.2–88.5%), representing the reference level prior to storage. After 24 h, fertilization rates showed significant differences among treatments (Fig. 3). Based on the estimated marginal means from the binomial model, fertilization rates were significantly higher at 4 °C (T1; 71.7%, 95% CI 64.5–77.9%) compared to both 2 °C (T2; 53.5%, 95% CI 49.2–57.7%) and 12 °C (T3; 55.0%, 95% CI 49.1–60.8%). Thus, deviations from 4 °C in either direction (cooling to 2 °C or warming to 12 °C) resulted in reduced fertilization capacity. Using 20 eggs per replicate resulted in wide uncertainty for these estimates, particularly where rates approached 50%. This limited the statistical power to detect moderate differences between treatments.

### Cold-bin sensitivity

When pooling 2 °C and 4 °C as a ‘cold bin’ (T1 + T2) and contrasting it with 12 °C (T3), fertilization was approximately + 7.6 percentage points higher in the cold bin (derived from model estimates). While this aggregate pattern supports the general benefit of cooling, it is important to note that this effect was primarily driven by the superior performance at 4 °C (71.7%), as the 2 °C treatment alone (53.5%) did not differ significantly from the 12 °C condition. This reinforces the practical recommendation to maintain maceration and short-term storage at stable low temperatures, ideally closer to 4 °C, to preserve fertilizing capacity.

### Association between flow cytometric biomarkers, motility parameters and fertilization rate

**Fig. 4:**
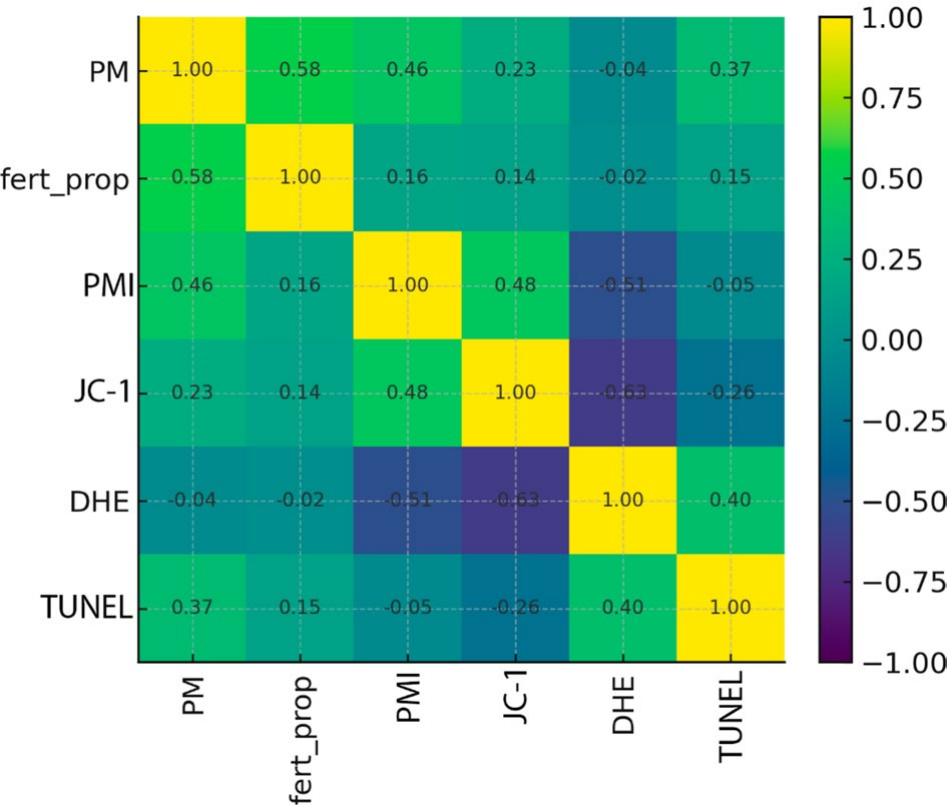
Spearman correlation heatmap (day + 1; T1–T3; male × treatment level). The color scale encodes ρ (positive in green/yellow, negative in blue/purple). Variables: PM = progressive motility; fert_prop = fertilization proportion; PMI = % SYTOX-negative (viable); JC-1 = ΔΨm (% red population/aggregates); DHE = % high; TUNEL = % TUNEL-positive. Cell labels show ρ. Correlations are associative; bootstrap 95% CIs and Holm-adjusted *p* values are provided in Supplementary Table S-Corr.

Associations (Spearman, day + 1; T1–T3). Overall, pairwise associations were modest once we restricted the analysis to day + 1 and adjusted for multiple testing. A moderate positive association was observed between progressive motility and fertilization (*ρ* = 0.58, Holm-adjusted *p* = 0.0119). At the cellular level, DHE (superoxide) showed negative associations with JC-1 (ΔΨm; red population) (*ρ* =  − 0.63, Holm-adjusted *p* = 0.0025) and with PMI (% SYTOX-negative) (*ρ* =  − 0.51, Holm-adjusted *p* = 0.0469). Other pairs were weak or non-significant after adjustment (Fig. 4). These associations are non-causal and are reported with bootstrap 95% CIs and multiple-testing corrections in Table S-Corr.

## Discussion

The results of this study indicate that the temperature during post-maceration storage in sex-reversed rainbow trout (*Oncorhynchus mykiss*) is associated with differences in sperm functional readouts after 24 h of storage, particularly in mitochondrial status (ΔΨm), oxidative stress (DHE, superoxide), and plasma membrane integrity (% SYTOX-negative). Although reproductive protocols generally emphasize semen storage and activation, less attention has been paid to temperature during gonadal maceration. Our results highlight that thermal management during this 24 h handling stage is a practical consideration for the production of monosex populations in aquaculture.

A key observation was the marked reduction in the proportion of JC-1 aggregate population (indicating lower ΔΨm) at the higher storage temperature (12 °C, T3), a pattern consistent with impaired mitochondrial bioenergetics. Mitochondria supply ATP for flagellar motility in fish sperm^9,24^; accordingly, lower mitochondrial functional status has been associated with shorter motility duration and reduced fertilization performance in salmonids^17,22,25^. Our results align with reports in trout and other salmonids where mitochondrial dysfunction, under cryopreservation, oxidative stress, or senescence, coincides with marked declines in sperm performance^17,22,25^.

In addition to the lower ΔΨm, exposure to 12 °C (T3) showed the highest proportion of DHE-high sperm (% DHE-high; superoxide). Elevated superoxide is associated with lipid peroxidation, DNA damage, and apoptotic pathways in fish sperm^21,26^. In our data, higher DHE in T3 coincided with the largest declines in motility parameters (VCL, VSL, VAP) and with lower plasma membrane integrity (% SYTOX-negative), consistent with a greater vulnerability of semen from sex-reversed males to warmer post-maceration handling^27,28^.

Plasma membrane integrity (PMI; % SYTOX-negative) is a well-established viability marker, reflecting the structural and functional integrity of sperm^29,30^. In our dataset, PMI was lowest at 12 °C (T3) after 24 h, aligning with reports that testicular sperm from sex-reversed males are susceptible to sublethal injury under suboptimal temperature and handling^31,32^. By contrast, DNA fragmentation (TUNEL; % TUNEL-positive) remained stable across treatments, which may reflect the short exposure window and/or pre-fertilization repair capacity.

Consistent with the functional readouts, statistical analyses showed significant differences in both total and progressive motility across treatments. Spermatozoa at 12 °C (T3) displayed significantly lower motility compared to the 4 °C (T1) treatment, a pattern consistent with reduced performance under warmer post-maceration handling. Kinematic values at 2 °C (T2) were generally intermediate or comparable to 4 °C (T1). However, the significantly lower velocities observed at 12 °C (T3) support integrating classical CASA kinematic metrics (VCL, VSL, VAP; TM/PM) with cellular biomarkers (ΔΨm; DHE; % SYTOX-negative) to obtain a more holistic assessment of sperm quality in sex-reversed rainbow trout.

Biomarkers such as ΔΨm and superoxide production (SYTOX/DHE) are established indicators of sperm quality in salmonids^2,9,26^. In this study, Spearman correlations revealed moderate associations, specifically between DHE and JC-1 (*ρ*≈ − 0.63) and DHE and PMI (*ρ*≈ − 0.51; both Holm-adjusted *p* < 0.05), while progressive motility and fertilization showed a positive association (*ρ*≈0.58; Holm-adjusted *p*≈0.012). Other pairs were weak/non-significant. These are associative readouts, reported with bootstrap 95% CIs and multiplicity adjustment (Table S-Corr). While direct correlations between mitochondrial status and individual kinematic parameters were moderate in this dataset, the significant associations between oxidative stress (DHE) and ΔΨm (*ρ*≈ − 0.63) suggest that these biomarkers serve as relevant contextual measures of cellular physiological status during post-maceration handling.

These findings contrast with previous studies in cryopreserved or senescent sperm of *Salmo salar* and *O. mykiss*, where strong negative correlations were reported between DHE (superoxide) and motility/fertilization, together with positive associations between mitochondrial status (ΔΨm) and overall sperm performance^17,26^. Such differences may reflect the specific physiological state of testicular spermatozoa under acute post-mortem storage conditions, where compensatory cellular mechanisms transiently buffer oxidative or mitochondrial dysfunction before these effects are detectable at the kinetic or fertilization level.

While the correlations in this specific dataset were moderate, these parameters remain sensitive indicators of cellular stress. Future investigations with larger cohorts and longitudinal measurements could further refine the use of these biomarkers to characterize sublethal responses in sex-reversed males.

Much of the salmonid literature addresses storage or activation temperature; here we focus on post-maceration handling in sex-reversed males as a distinct workflow step. Our results indicate that storage temperature following tissue disruption is associated with differences in sperm functional integrity, underscoring the need to explicitly control this step.

Since sex-reversed males are sacrificed for semen collection, optimizing the use of each individual is crucial. In our dataset, post-maceration handling at 4 °C (T1) was associated with the best preservation of motility, mitochondrial status (ΔΨm), and significantly higher fertilization rates compared to both 2 °C (T2) and 12 °C (T3). Although the cold-bin analysis (pooling T1 + T2 vs T3) suggests a general benefit of preventing warming, the specific drop in fertilization at 2 °C indicates that ‘colder’ is not always better for functional capacity. Therefore, as a practical intervention, maintaining tools, surfaces, and homogenates at stable temperatures near 4 °C could maximize egg fertilization and fry quality, lowering broodstock requirements and contributing to favorable economic and environmental outcomes.

Overall, this study frames post-maceration handling temperature as a critical early-handling variable that is associated with differences in semen quality in sex-reversed males. While the direct association between functional readouts and fertilization outcomes may be complex under acute stress conditions, the biological relevance of mitochondrial status (ΔΨm) and superoxide (DHE) remains supported by prior work, as indicators of cellular physiological status. In practical terms, our results support maintaining post-maceration handling at stable low temperatures, ideally close to 4 °C, to avoid the deleterious effects of warming (12 °C) or excessive cooling (2 °C). This focus on protocol optimization, particularly temperature control during the post- processing stage, is a key strategy for reproductive success in *Oncorhynchus mykiss*. Future studies should refine handling workflows, integrate advanced biomarkers into routine QA/QC.

### Limitations found

Fertilization was estimated from subsamples of 20 eggs per replicate; accordingly, we report binomial 95% CIs and acknowledge higher uncertainty than larger subsamples would yield. Eggs were pooled by female/lot, so fertilization reflects a grouped denominator rather than per-egg independence. Cytometry endpoints are proportions by gating (fluorescence proxies, not calibrated intensities): PMI = % SYTOX-negative, DHE = % DHE-high, TUNEL = % TUNEL-positive, and JC-1 (ΔΨm) = % red population (aggregates). Some endpoints were not strictly contemporaneous; therefore associations (JC-1/PMI/DHE → motility → fertilization) are interpreted as non-causal. Raw gate images from the original runs were not archived; instead, we provide a representative gating/QC schematic (Fig. S-Gating). The use of a high, non-limiting sperm dose (~ 15 × 10^6^ spermatozoa per egg) may have partially masked subtle differences in sperm quality between treatments. Future investigations using limiting sperm doses might reveal stronger associations between these cellular biomarkers and fertilization success. Findings are limited to sex-reversed rainbow trout under this post-maceration handling workflow (0–4 °C recommended).

## Conclusion

This study demonstrates that temperature during the 24 h post-maceration storage period is associated with differences in sperm quality in sex-reversed rainbow trout (*O. mykiss*), observable before fertilization with respect to CASA motility, mitochondrial status (ΔΨm), oxidative status (DHE), and plasma membrane integrity (% SYTOX-negative). While JC-1 and DHE showed weak to moderate correlations with reproductive outcomes under acute thermal exposure, their physiological relevance remains supported by prior work and argues for their context-dependent diagnostic use alongside CASA and binomial fertilization (k/n with CIs). Flow-cytometry applications in sex-reversed rainbow trout under post-mortem, thermally stressful conditions are still scarce, underscoring operational vulnerability to handling temperature. Our per-treatment contrasts and the cold-bin sensitivity (2–4 °C vs 12 °C) suggest that maintaining post-maceration handling at 0–4 °C may be beneficial. Future work should validate these readouts under routine hatchery conditions using larger cohorts, repeated time points, and improved temperature verification, such as in-sample probes and continuous logs. Furthermore, research should explore scalable strategies to limit thermal stress during handling. Associations are interpreted non-causally and are limited to sex-reversed males within this maceration workflow.

## Materials and methods

### Ethics approval

The animal experiment protocol was reviewed and approved by the Animal Ethics Committee of the Catholic University of Temuco (approval number CEIUCT 0,420,001/23). This study followed all guidelines described in the “Guide for the Care and Use of Laboratory Animals” (National Research Council, 2011).

### Experimental design and animals

The study was conducted at Huililco Fish Farm Ltda. (Pucón, La Araucanía Region, Chile) during the 2023 reproductive season. Ten sex-reversed male rainbow trout (*Oncorhynchus mykiss*), 2 years old in their first seasonal maturation (~ 2 y), were randomly selected from the available stock of mature males at the center. Mean body mass was 1.07 ± 0.13 kg and total length 43 ± 1.4 cm. Fish were held in 33 m^3^ raceways (open flow ~ 5 L s⁻^1^; density ~ 24 kg m⁻^3^); mean water temperature was 10.1 °C; dissolved O_2_ was ~ 10.1 mg L⁻^1^ at inlet and ~ 7.5 mg L⁻^1^ at outlet. Photoperiod was 18:6 h (light:dark) in summer and 4:20 h in winter.

An intramale model was implemented, using repeated measurements to evaluate the effects of post-maceration storage temperature and time on sperm quality. For each male, both testes were collected, macerated, and the homogenate was divided into four equal aliquots, one per treatment. The treatment structure was: T0 = 4 °C, 0 h; T1 = 4 °C, 24 h; T2 = 2 °C, 24 h; T3 = 12 °C, 24 h. This pairing enables within-individual contrasts and reduces inter-individual variability. The experimental design intended to expose all males to all treatments; any missing values or non-contemporaneous endpoints per individual are accounted for in the statistical models (GLMM) used for inference.

### Sampling, transport, and temperature control

Transport was performed at 4 °C for approximately 1 h 30 min using a thermoelectric portable cooler with an internal digital display. In the laboratory, aliquots were stored for 24 h at 4 °C (laboratory refrigerator; T1), 2 °C (refrigerated centrifuge used as a static chamber; T2), or 12 °C (lidded thermoblock; T3) before analysis, all in the dark and without agitation. Temperatures were monitored and verified using the digital display on each storage device (refrigerator, centrifuge chamber, thermoblock), which recorded the internal air temperature. Measurements were recorded at the beginning and end of the 24 h storage period (and midway through the period when possible). The recorded temperatures remained stable and within ± 0.5 °C of the target nominal temperatures (2, 4, and 12 °C) throughout the storage period. A limitation of this method is that it reflects the ambient temperature of the chamber and not the temperature of the sample medium itself.

### Sperm collection and handling

Fish were euthanized with MS-222. Testes were aseptically removed, weighed, and macerated with sterile scalpels on chilled plates maintained at ~ 4 °C to preserve gamete viability during tissue disruption. The homogenate was filtered through a sterile 30–50 µm mesh and diluted 1:1 with Storfish® diluent. The diluted suspension was transported to the Catholic University of Temuco at ~ 4 °C. Upon arrival at the laboratory (Time 0), the sample was immediately divided into four equal aliquots**.** This ensured that transport conditions were identical for all The T0 aliquot was processed for fertilization and analysis immediately upon arrival, using the same pooled batch of oocytes that was subsequently used for the T1–T3 fertilizations 24 h later.

For treatments, samples were stored under controlled temperatures (2, 4, 12 °C) for 24 h (dark, no agitation). The control (T0) was processed immediately upon arrival/dilution (0 h at 4 °C). These temperatures bracket cold (0–4 °C, typical for quality preservation) and a warmer condition (12 °C) used in short-term experimental designs (*Danio rerio, Salmo salar*) to elicit thermal effects on motility, mitochondrial status and DNA integrity^10,27,33^.

### Operational definition of treatments and inferential scope

Upon arrival, each male’s homogenate was subdivided into identical aliquots. T0 aliquots were processed immediately (0 h post-arrival at 4 °C). The remaining aliquots were stored for 24 h at 4 °C (T1), 2 °C (T2), or 12 °C (T3) and then processed. Thus, all samples experienced the same 4 °C transport, and the T0 vs T1–T3 contrast isolates post-arrival storage effects (time/temperature) while holding transport constant. For each treatment, all males were run in the same analytical session (T0 on the day of arrival; T1–T3 after 24 h of storage). For statistical inference and lettering in figures, T0 is shown descriptively and not included in between-treatment tests; the primary contrasts are among T1–T3 (day + 1). The complete dataset for all male-treatment-endpoint combinations was incorporated into the generalized linear mixed-effects models (GLMM) to account for any missing observations. Sensitivity results for cold bins (T1 + T2) vs T3 are provided in Supplementary Fig. S-ColdBins.

### Sperm quality parameters

#### Motility

Sperm motility was quantified with a computer-assisted sperm analysis system (CASA; Sperm Motility Tracker v2.0, IST Reproductive Technologies) on a microscope with a 10 × objective, digital camera (30 fps, 720 × 576 px), and a temperature-controlled stage set at 4 °C. Semen (0.5–1.0 µL) was activated with 5 µL PowerMilt® activator on chilled slides and loaded into a fixed-depth CASA chamber (chamber depth and all acquisition/analysis settings in Supplementary Table S1). For each sample, we performed three independent activations (technical triplicates); for each activation, one non-overlapping field was recorded at 5 s post-activation, and ≥ 100 sperm tracks were quantified per field. Summary metrics for a sample were computed as the mean of the three activations.

Kinematic parameters included VCL, VSL, and VAP (µm s⁻^1^), and the dimensionless ratios STR (100 × VSL/VAP) and LIN (100 × VSL/VCL). Following common CASA practice in salmonids and to ensure reproducibility within this system, total motility (TM) was defined as the fraction of cells with VAP ≥ 15 µm s⁻^1^, and progressive motility (PM) as the fraction with STR ≥ 45% and VAP ≥ 30 µm s⁻^1^. Because progressive trajectories in salmonids are often curvilinear, LIN is reported descriptively but not used as a hard cut-off. All details on segmentation thresholds, minimum object size, linking radius/maximum jump, smoothing, minimum track length, frame rate, resolution, chamber depth, spatial calibration, and stage temperature are provided in Supplementary Table S1. Sperm concentration was measured with a Neubauer chamber (modified from ref.^34^).

### Plasma membrane integrity (PMI)

The viability and PMI of fish spermatozoa were assessed using a dual fluorescent staining protocol with SYBR-14 and propidium iodide (PI), adapted from protocols validated for teleost species. A sperm suspension was prepared at 4 × 10^6^ cells/mL in phosphate-buffered saline (PBS, pH 7.4). The sample was incubated for 10 min at 4 °C in the dark with 1 μL of SYBR-14 and 1 μL of PI, both from the LIVE/DEAD® Sperm Viability Kit (Invitrogen, Thermo Fisher Scientific, Waltham, MA, USA). Following incubation, samples were analyzed immediately by flow cytometry. PMI is reported as “% SYTOX-negative (intact)” obtained by flow-cytometric gating. Viable spermatozoa (SYBR-14⁺/PI⁻, fluorescing green) were distinguished from non-viable spermatozoa (SYBR-14⁻/PI⁺, fluorescing red) based on fluorescence signals detected at 530 ± 15 nm (SYBR-14) and > 670 nm (PI)^35^.

### Mitochondrial status (JC-1, ΔΨm indicator)

ΔΨm was evaluated with JC-1 (MitoProbe™ JC-1 Kit), adapted for fresh fish sperm. Cells were incubated with JC-1 (manufacturer’s recommended working concentration) for 10 min at 4 °C in the dark and read immediately^9^. The endpoint is expressed as the “% JC-1 red population (aggregates)”. A higher percentage of cells in this population is interpreted as indicating a higher mitochondrial membrane potential (ΔΨm).

### Production of superoxide anion (O_2_⁻, SYTOX/DHE)

To evaluate cytoplasmic oxidative stress, the production of superoxide anion (O_2_⁻·) was measured using the fluorescent probe dihydroethidium (DHE) in combination with SYTOX® Green to exclude non-viable cells. The protocol was adapted and optimized for application in fish sperm cells^36^. Briefly, sperm samples were incubated with DHE at a final concentration of 2.5 µM and SYTOX® Green at 0.5 µM for 15 min at 10 °C in the dark. Results are reported as “% DHE-high (superoxide) among live (SYTOX-negative) sperm” based on a gate defined from controls.

### DNA fragmentation (TUNEL)

DNA fragmentation was analyzed using the In Situ Cell Death Detection Kit—Fluorescein (Roche Diagnostics, Mannheim, Germany), applying a modified protocol^37^. Briefly, spermatozoa were fixed and permeabilized to enable incorporation of labeled nucleotides at DNA strand breaks. The samples were incubated with the TUNEL reaction mixture at 37 °C in the dark, as recommended by the manufacturer. Following incubation, cells were washed and resuspended for immediate analysis by flow cytometry. DNA fragmentation was quantified as the percentage of TUNEL-positive sperm cells. All measurements were performed in triplicate under standardized experimental conditions.

### Flow cytometry acquisition and QC

Data were acquired on a BD Accuri™ C6 Plus with standard optics (488 nm blue and 640 nm red lasers). Typical detectors used were FL1 533/30 nm (green; SYTOX, JC-1 monomers), FL2 585/40 nm (orange; DHE, JC-1 aggregates) and FL3 670 LP (red; PI). At least 10,000 events per sample were collected; fluorescence signals were amplified and recorded using logarithmic scales. Acquisition was performed on chilled holders/stage (~ 4 °C). Gating/QC sequence: P1 (FSC-A vs SSC-A) to exclude debris → singlets (FSC-A vs FSC-H) to exclude doublets → live/dead by SYTOX → endpoint-specific gate (JC-1 red, DHE-high, TUNEL-positive). Thresholds were set against unstained/FMO controls and held constant within a run. Spectral compensation followed the lab’s standard matrix. A representative gating schematic is shown in Fig. S-Gating. All signals were collected on logarithmic scales. Instrument settings, gating strategies, and sample handling followed standardized protocols validated for fish sperm analysis^26^.

### Fertilization assay

Fertilization capacity was assessed using oocytes obtained from a pool of five mature females to minimize variability associated with post-ovulatory aging. Oocytes were retrieved, macroscopically assessed for maturity/overmaturity, and divided into two batches. One batch was used for T0 fertilization assays immediately after sperm samples arrived at the laboratory. The second batch was stored for 24 h at 4 °C in a humidified atmosphere before being used for T1–T3 fertilization assays. This approach minimized variability, although potential effects of post-ovulatory aging during storage cannot be fully excluded. For each male and treatment (T0–T3), fertilization was performed individually using that male’s semen aliquot. Twenty (20) eggs were fertilized in triplicate (technical replicates) per male × treatment. A high, non-limiting sperm dose (~ 15 × 10^6^ spermatozoa per egg) was used to ensure fertilization in testicular macerates. While this dose was chosen to guarantee measurable reproductive outcomes, we acknowledge that such non-limiting conditions may partially mask subtle physiological differences in sperm quality between treatments.

For each replicate, a known semen volume (µL) was dispensed, computed from the sperm concentration (sperm/µL) measured by Neubauer chamber in the same sample. When relevant, we also report the achieved ratio (based on the applied volume). Per-male calculations are provided in Supplementary Table S-Sperm: Egg. Fertilizations were performed on chilled plates at ~ 4 °C. Gametes were gently mixed, activation was initiated with hatchery water at 4 °C, and eggs were swirled for ~ 60–90 s before rinsing. Eggs were then transferred to incubation trays at 4 °C and scored at the 4-cell stage (~ 10–12 h) under a stereomicroscope. No embryo transport was involved after fertilization.

### Fertilization (k of n)

Fertilization was analysed as a binomial response using a mixed-effects logistic model (GLMM, logit link) with treatment as a fixed effect and male as a random intercept (within-male pairing across treatments). We report odds ratios (ORs) with 95% CIs and Holm-adjusted p values for pairwise contrasts (T3 vs T1, T3 vs T2). In addition, we present effect sizes in percentage points (pp) as marginal mean differences with bootstrap 95% CIs. As a robustness check, we fitted a GEE (binomial, logit; exchangeable working correlation) with male as the clustering factor; inferences were consistent.

### Flow-cytometry endpoints and CASA motility (proportions without denominators)

Endpoints expressed as percentages by gating (PMI = % SYTOX-negative; DHE = % high; TUNEL = % positive; JC-1 (ΔΨm) = % red population) and motility proportions (TM/PM) were modelled as proportions using a beta mixed model (GLMM, logit link) with treatment fixed and male random. Values at the boundaries were handled with the Smithson–Verkuilen adjustment to (0,1). Where beta assumptions were sensitive to boundary mass, we corroborated results with a fractional-logit model and cluster-robust (male-level) standard errors. We report marginal means (in %), 95% CIs, and Holm-adjusted pairwise tests among T1–T3.

### Multiple testing

For within-endpoint pairwise comparisons we control family-wise error with Holm; BH/FDR values are also reported in Supplementary Tables.

### Associations

To evaluate non-causal associations among biomarkers, motility and fertilization we computed Spearman correlations on day + 1 data at the male × treatment level (T1–T3). We provide bootstrap 95% CIs and Holm-adjusted p values (Table S-Corr).

### Sensitivity (“Cold Bins”)

Because 2 °C and 4 °C are both “cold”, we conducted a within-male sensitivity comparing cold bins (T1 + T2) vs warm (T3) using paired Wilcoxon tests (fertilization and key cytometry endpoints) and report 95% Wilson CIs per male (Fig. S-ColdBins; Table S-ColdBins).

### Statistical analysis

All analyses were performed in R 4.3.2. Between-treatment inference was restricted to day + 1 (T1–T3); T0 (4 °C, 0 h) is shown descriptively (†) to avoid confounding day vs treatment.

### Model diagnostics and goodness-of-fit

To ensure the robustness of the statistical inferences, all models underwent a comprehensive diagnostic evaluation. Fertilization success (number of fertilized eggs out of n per replicate) was analyzed with generalized linear models (GLM; binomial family, logit link; glm() in R). Because multiple observations were obtained from the same males, we reported cluster-robust standard errors by male using the *sandwich* and *lmtest* packages (male as the clustering variable) to account for within-subject correlation. Convergence was verified (no warnings, typical IRLS convergence within a few iterations), and model adequacy was assessed using log-likelihood, AIC, BIC, McFadden’s pseudo-R^2^, and the Pearson χ^2^ divided by residual degrees of freedom (χ^2^/df) as an overdispersion check. Residual structure was examined via deviance-residual histograms, residuals-versus-fitted plots, and QQ-plots generated with performance and DHARMa.

For flow-cytometry proportions (PMM, PMI, DHE, and DNA fragmentation) and motility percentages (total and progressive), we fitted fractional-binomial GLMs (binomial family, logit link in glm()), applying a small Smithson–Verkuilen adjustment to keep proportions within the open (0,1) interval when needed. As above, we used cluster-robust SEs by male, and evaluated log-likelihood, AIC/BIC, McFadden’s pseudo-R^2^, χ^2^/df, and the same residual diagnostics (deviance residuals, residuals-vs-fitted, QQ-plots). Estimated marginal means (± 95% CI) were obtained with emmeans for treatment contrasts, and effect magnitudes were expressed as odds ratios or marginal probability differences derived from the fitted models.

For CASA kinematics (VCL, VSL, VAP), we used linear mixed-effects models (lme4::lmer) on log1p-transformed responses to stabilize variance, with a random intercept for male (model: log1p(response) ~ treatment + (1|male)). Diagnostics included convergence status, AIC/BIC, log-likelihood, the intraclass correlation coefficient (ICC), Shapiro–Wilk tests of residuals, and residual plots (histograms, residuals-vs-fitted) plus QQ-plots. Where the random-effects covariance was close to zero (singular-fit warnings), this was interpreted as negligible between-male variance rather than model failure, and fixed-effect inferences remained valid.

Across all analyses we adopted two-sided α = 0.05 after verification of model assumptions and residual distributions. Computations were conducted in R 4.3.2 using *stats*, *sandwich, lmtest, emmeans, lme4/lmerTest, performance, and DHARMa*.

## Supplementary Information

Supplementary Information 1.

Supplementary Information 2.

Supplementary Information 3.

Supplementary Information 4.

Supplementary Information 5.

Supplementary Information 6.

Supplementary Information 7.

## Data Availability

The datasets used or analyzed during the current study are available from the corresponding author on reasonable request.
