# Supplementary figures and images for: Post-maceration storage temperature affects sperm quality and fertilization in sex-reversed rainbow trout
Source: Sci Rep. 2026 Apr 11;16:17081. doi: 10.1038/s41598-026-46962-4 (PMC13230734; doi:10.1038/s41598-026-46962-4)

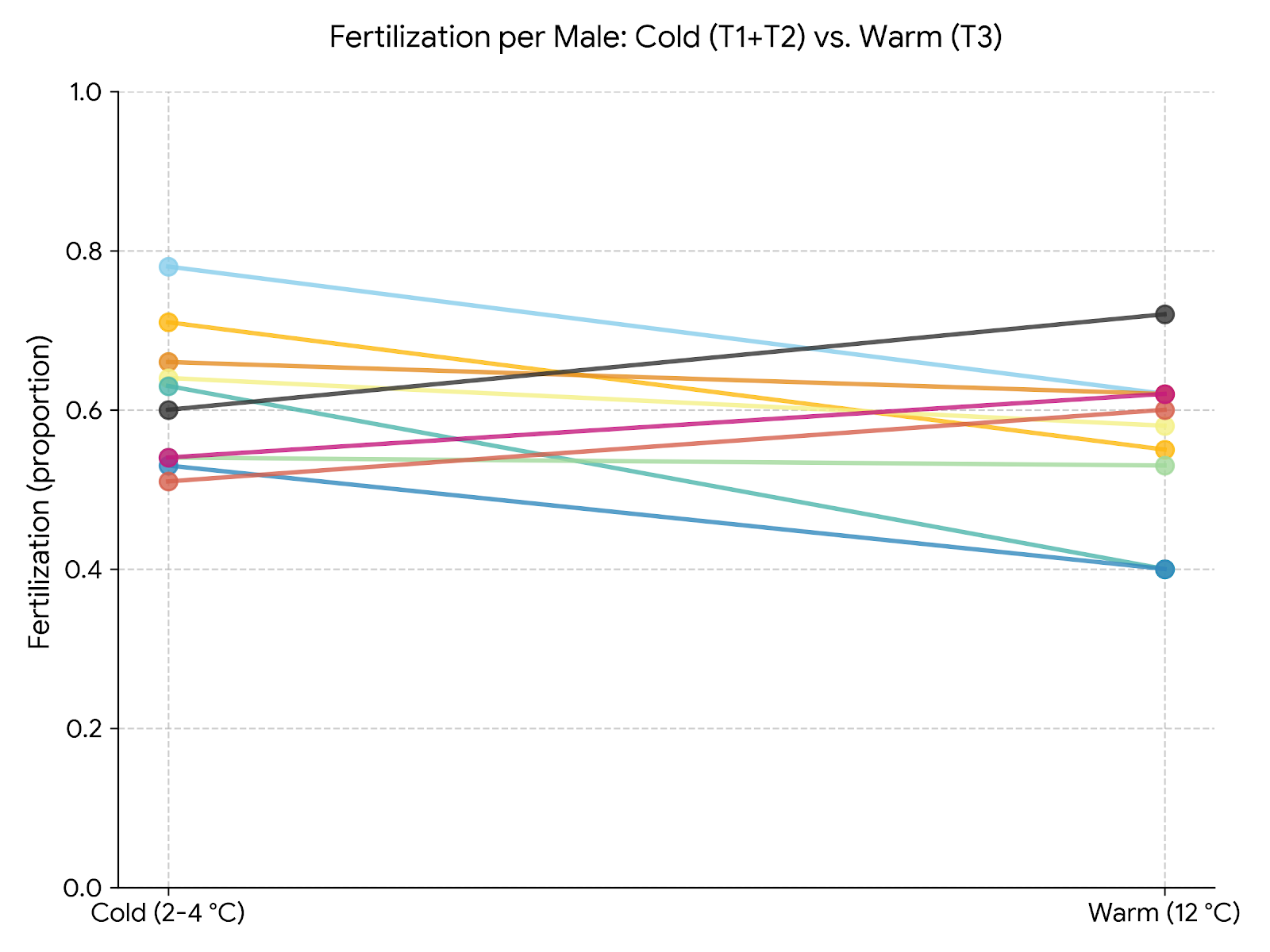

Supplement: Supplementary file 2 — Supplementary Information 2. [file 41598_2026_46962_MOESM2_ESM.png]
