# Supplementary material for: Post-maceration storage temperature affects sperm quality and fertilization in sex-reversed rainbow trout
Source: Sci Rep. 2026 Apr 11;16:17081. doi: 10.1038/s41598-026-46962-4 (PMC13230734; doi:10.1038/s41598-026-46962-4)
Supplement: Supplementary file 3 — Supplementary Information 3. [file 41598_2026_46962_MOESM3_ESM.docx]

**Supplementary Table S1. CASA configuration and thresholds (Sperm Motility Tracker 2)**

*This table reports the exact acquisition and analysis settings used for CASA with Sperm Motility Tracker 2 (SMT2). The parameters were kept constant across all recordings.*

| Parameter | Value |
| --- | --- |
| Software | Sperm Motility Tracker 2 (SMT2) |
| Objective | 10× |
| Chamber depth | 10 µm |
| Frame rate | 30 fps |
| Video resolution | 1920 × 1080 px |
| Temperature during analysis | 4°C |
| Post-activation analysis window | 5.0 s |
| Spatial calibration | 100 µm = 107 px |
| Threshold (binarization) | 20 |
| Object area (min – max) | 15 – 150 px² |
| Grayscale structuring element (diameter) | 11 px |
| Binary structuring element (diameter) | 5 px |
| Max search radius (linking) | 25 px |
| Moving-average window for VAP/ALH | 5 values |
| Minimum track length | 25frames |
| Total motility (TM) | VAP ≥ 15 µm s⁻¹ |
| Progressive motility (PM) | STR ≥ 45% AND VAP ≥ 30 µm s⁻¹ |
| Speed classes (descriptive) | VAP: 15–30, 30–50, ≥50 µm s⁻¹; Static: VAP < 15 µm s⁻¹ |
